# Special Resins for Stereolithography: In Situ Generation of Silver Nanoparticles

**DOI:** 10.3390/polym10020212

**Published:** 2018-02-22

**Authors:** Gabriele Taormina, Corrado Sciancalepore, Federica Bondioli, Massimo Messori

**Affiliations:** 1 Department of Engineering and Architecture, University of Parma, Parco Area delle Scienze 181/A, 43124 Parma, Italy; gabriele.taormina@studenti.unipr.it (G.T.); federica.bondioli@unipr.it (F.B.); 2 INSTM, National Interuniversity Consortium of Materials Science and Technology, Via Giusti 9, 50121 Florence, Italy; corrado.sciancalepore@instm.it; 3 Department of Engineering “Enzo Ferrari”, University of Modena and Reggio Emilia, Via Pietro Vivarelli 10/1, 41121 Modena, MO, Italy

**Keywords:** 3D printing, stereolithography, acrylic resin, nanocomposite, silver nanoparticles, in situ generation

## Abstract

The limited availability of materials with special properties represents one of the main limitations to a wider application of polymer-based additive manufacturing technologies. Filled resins are usually not suitable for vat photo-polymerization techniques such as stereolithography (SLA) or digital light processing (DLP) due to a strong increment of viscosity derived from the presence of rigid particles within the reactive suspension. In the present paper, the possibility to in situ generate silver nanoparticles (AgNPs) starting from a homogeneous liquid system containing a well dispersed silver salt, which is subsequently reduced to metallic silver during stereolithographic process, is reported. The simultaneous photo-induced cross-linking of the acrylic resin produces a filled thermoset resin with thermal-mechanical properties significantly enhanced with respect to the unfilled resin, even at very low AgNPs concentrations. With this approach, the use of silver salts having carbon-carbon double bonds, such as silver acrylate and silver methacrylate, allows the formation of a nanocomposite structure in which the release of by-products is minimized due to the active role of all the reactive components in the three dimensional (3D)-printing processes. The synergy, between this nano-technology and the geometrical freedom offered by SLA, could open up a wide spectrum of potential applications for such a material, for example in the field of food packaging and medical and healthcare sectors, considering the well-known antimicrobial effects of silver nanoparticles.

## 1. Introduction

Additive manufacturing (AM) is a group of technologies that are revolutionizing many aspects of the design and production processes behind the realization of an increasing number of products. This disruptive new way to realize three-dimensional (3D) objects has had a heavy impact on a lot of industrial sectors [1], also thanks to the vivid interest inspired in different research fields from engineering to biomedical, passing through many other relevant disciplines such as cultural heritage [2].

The main advantage of this innovative way to produce is the capability to reach high geometrical complexity, maintaining the appropriate accuracy, at a low cost. In this respect, AM technologies are sometimes called “free form fabrication” techniques. In some cases, AM is the only way to produce very complex parts, other times it is a valid aid to reduce the number of different components in a complex object which traditionally has to be assembled from several parts. The advantages in the prototyping phase are enormous, the time and cost to realize different prototypes to study their properties, functionalities, and aspects are significantly reduced. AM can also offer advantages in the reduction of the cost to customize products, with respect to traditional production methods [3].

Inside the wide AM family there are several different technologies, and even if some new technologies are currently under development, many more are already available at a sufficiently level of technological maturity, as confirmed by the increasing percentage of AM based industrial productions [4].

Among the 3D printing technologies developed for polymeric materials, a possible classification can start from the different materials form and state. Some of the technologies are able to process powders and can create an object by depositing a binder to guarantee the cohesion of powder layers (binder jetting) or by selectively sintering or melting them (powder bed fusion). Some other technologies start from solid-form materials (e.g., pellets, wires), which must be melted before being selectively deposited to form each layer of the object (material extrusion). Other different technologies are able to process liquid-form material to locally deposit and cure it (material jetting) or to selectively cure it from a liquid vat (vat photo-polymerization). Vat photo-polymerization comprehends two different technologies, one based on a vector scanning curing provided by a laser (stereolithography, SLA) and the other on a mask projection curing system (digital light processing, DLP). One of the main drawbacks of this kind of technology is the increase of viscosity and the possible sedimentation phenomena that tend to strongly limit the use of filled photo-curable resins in both stereolithography (SLA) and DLP technologies [5,6,7,8].

SLA is one of the most interesting technology among the wide AM family, because it allows one to reach the highest levels of accuracy combining the advantages of starting from a liquid base material with the vector scanning driven modality that the laser adopts to cure it. The only higher limitations to the accuracy are the laser spot size and the z axis step increase of each layer [9].

The approach on composite materials is the will to take advantage of useful properties of different materials, while trying to limit their singular drawbacks. Adding a nanometric filler can significantly increase the properties of the resulting nanocomposites mainly due to the higher surface-to-volume ratios of nanofillers that promotes surface interactions, affecting positively the properties of the nanocomposites starting from very low loadings [10,11,12]. The great advantage deriving from the combination of AM and nanotechnology to produce nanocomposites can easily explain the numerous articles that have been published on the subject. Often authors tried to increase the mechanical properties of nanocomposites while understanding the correlation between filler content and nature with the variation in properties [5,7,13,14,15,16,17,18,19,20]). However, the increase in mechanical properties often brings a decrease in ductility and some work has been done to avoid it [5,16]. Though mechanical properties are the most studied, some work has been done also on attempting to increase electrical and thermal properties [7,14,21,22]. Researchers of many other application fields have been interested in combining nanocomposites with AM. Studies have been published on biomedical applications [23], radar absorbing materials [24], piezoelectric materials [25], the creation of metal coating layers for flexible substrates [26], the development of hydrogel nanocomposites as substrates for antibacterial uses [27], and the fabrication of printable elastic conductors [28], while some other researchers focused on better understanding all the technological aspects involved and develop new printing techniques [13,29,30,31].

In a previous paper, the feasibility of 3D printing of parts based on acrylic photo-curable formulations, containing silver nanoparticles (AgNPs) in situ generated by UV-induced reduction of silver acetate, has been reported [32]. This approach involves a simultaneous polymerization-reduction process, where the polymerization of acrylic monomers and the reduction of silver ions to metallic silver occur at the same time by means of the laser action of an SLA printer. However, in this context the acetate ions are not an integral part of the polymeric structure and, although they are compatible with the chemical environment of the polymer network, they are not chemically bound and can be released by the system under certain conditions. This aspect is common for all those silver salts, whose anions do not actively participate in the polymerization process. An undesirable behavior, especially if applications in the biomedical field are considered. In the present paper, photo-curable liquid formulations containing different types and amount of silver salts as metallic silver particle precursors were prepared and converted in a solid part by means of a SLA. The laser light activated simultaneous both the cross-linking of organic resin and the reduction of silver cations into metallic AgNPs, in order to obtain a homogeneous composite material in which the starting reactants are converted integrally into the final structure. In particular, silver acrylate and silver methacrylate salts were used as suitable silver salts. This type of salts, in addition to providing the silver cations for the AgNPs formation process, are able to react with the acrylic monomers during the radical polymerization step due to the presence of alkenyl groups, reactive towards the cross-linking reaction of acrylic resin. Moreover, the carboxylate groups present in the molecular structure of the precursor salt can coordinate the surface of the silver nanoparticles as capping agent, leading to an increase and optimization of the interface interaction [33,34]. In this way, all the initial components of the system become a constituent element of the cross-linked structure. The absence of unreacted components from the photo-curable formulation eliminates the possibility of undesired leaching and the presence of AgNPs assures a strong anti-bacterial activity, enhanced by the nanometric dimensions of the particles. The combination of these two aspects can easily promote the application of such a nanocomposite material to fields (e.g., food contact, healthcare, and medical field) that can benefit from its bactericidal properties, reducing the infection or contamination risks. As a matter of fact, the chemical and physical properties of silver nanocomposites have already promoted their use in many fields and SLA and AM, in general, can add to this practice the design freedom in the production of the 3D engineered structures with customized shape [35,36]. The morphology and the mechanical properties of the obtained filled polymers were investigated and discussed.

## 2. Materials and Methods

### 2.1. Materials

The starting monomers were commercially available acrylic resins provided by Allnex (Allnex Holding S.à.r.l., Grand Rue, Luxemburg), in particular an amine functional acrylic resin (Ebecryl 7100) and pentaerythritoltriacrylate (PETIA). Bis-(2,4,6-trimethylbenzoyl)-phenylphosphine oxide was used as photo-initiator and was purchased from BASF (Ludwigshafen am Rhein, Germany) under the trade name of Irgacure 819 (Ir 819). Two AgNPs precursors, silver acrylate (AgAcr) and silver methacrylate (AgMAcr), were synthesized from silver nitrate and sodium acrylate and methacrylate, respectively. All the chemicals were high purity reactants purchased from Sigma-Aldrich (Milan, Italy).

AgAcr was synthesized by mixing at room temperature silver nitrate and sodium acrylate water solutions. The salts were mixed in stoichiometric ratio and the exchange reaction was instantaneous. The precipitated AgAcr salt was washed once in water and twice in ethanol and ultracentrifuged (Neya 16, Remi Neya, India) at 12,000 rpm for 5 min each step. AgMAcr was synthesized in the same way by using sodium methacrylate instead of sodium acrylate.

### 2.2. Samples Preparation

**Scheme 1:**
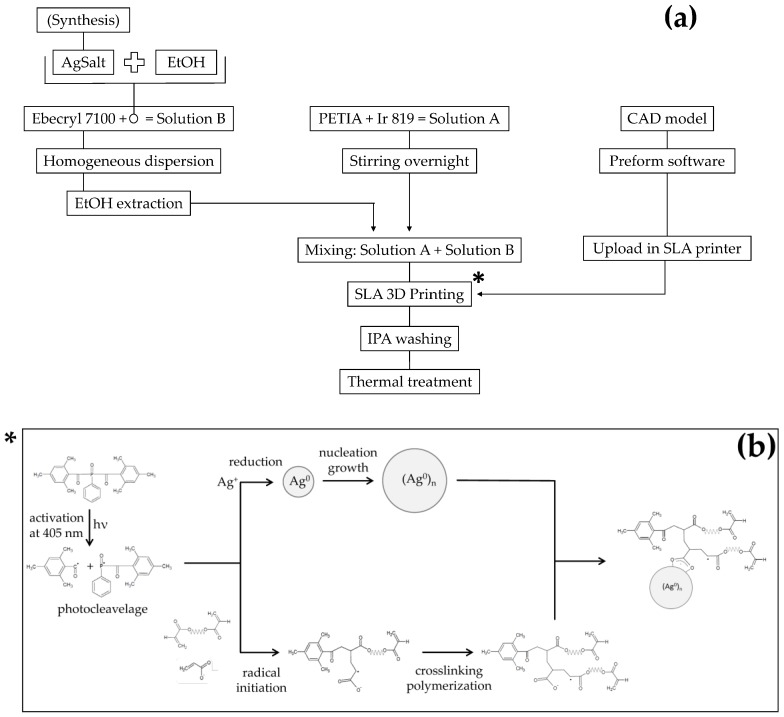
Schematic representation of materials preparation, three dimensional (3D) printing and post-curing steps (**a**) and expected reactions during the 3D printing process (**b**).

The photo-curable resin was obtained by mixing together two different mixtures (A and B) for a total of 100 g, shortly before the printing step, as reported in Scheme 1a. The A mixture was formed by 33 g of PETIA with the addition of 0.25 wt % of Ir 819 with respect to the total final mass. This mixture was mixed overnight until complete dissolution of the photo-initiator. The B mixture was formed by 67 g of Ebecryl 7100 and a suitable quantity of silver salt, necessary to obtain the required concentration of AgNPs in the final sample. To ease the dispersion in the resin, a small quantity of ethanol was added to the salt and removed afterwards by a dynamic vacuum treatment on the solution carried out at room temperature. A photo-curable resin formulation was also prepared without the presence of silver salts, as reference material.

The samples were coded “AgAcr *x*%” and “AgMAcr *x*%” for polymers containing AgNPs obtained from silver acrylate and silver methacrylate as precursors, respectively (*x* = 0.5, 1 and 2 corresponds to the nominal weight percent of AgNPs, by assuming a complete conversion of silver salt to metallic silver).

**Figure 1:**
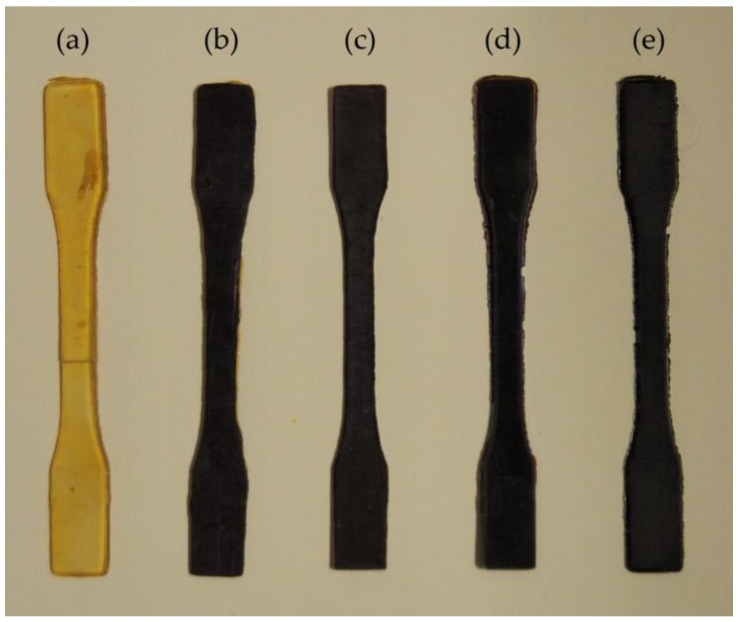
3D printed specimens: (**a**) unfilled; (**b**) 0.5% AgAcr; (**c**) 1% AgAcr; (**d**) 2% AgAcr; and (**e**) 1% AgMAcr.

A commercial inverted SLA printer (Form 2, Formlabs Inc., Somerville, MA, USA) was used to process the photo-curable formulations. The layer thickness was set to 50 μm. The CAD software SolidWorks (Dassault Systems SolidWorks Corporation, Waltham, MA, USA) was used to design the model for the specimens and the CAD file was then converted to an .stl file to be processed by the printer software (Preform 2.3.3, Formlabs, Somerville, MA, USA, 2016). The printed specimens were dumbbell-shaped according to the 1BA specimen type as indicated in the ISO 527-2 technical standard. The specimens were produced with the stacking direction along the specimen thickness and used for the subsequent structural and functional characterizations (Figure 1). The printing process time was between 1.5 and 2 h and the photo-curable formulation was observed to be stable for the whole period without any phase separation or side-reactions. The expected reactions occurring during the 3D printing step are shown in the Scheme 1b. The reactions of silver ions reduction and radical polymerization take place simultaneously, both activated by UV radiation, and lead to the formation of a homogeneous composite. In this system, the acrylate and methacrylate ions become part of the polymeric cross-linked structure and at the same time can coordinate AgNPs by means of the carboxylate groups, making interface interactions between filler and polymer matrix more effective.

At the end of the printing step, the specimens were detached from the build platform and rinsed in *iso*-propylic alcohol (IPA) for 20 min to fully remove all the residues of unreacted resin. Finally, a thermal post-curing treatment was carried out at 90 °C for 1 h, to complete the polymerization process partially inhibited by the addition of the silver salt (see DSC results reported below).

### 2.3. Samples Characterization

Differential scanning calorimetry (DSC) analysis was carried out at a heating rate of 10 °C/min from −10 to 200 °C in nitrogen atmosphere (DSC, TA2010, TA Instruments, New Castle, DE, USA).

Transmission electron microscopy (TEM) was carried out (Tecnai 12 Gspirit electron microscope, FEI Company, Hillsboro, OR, USA) by using an accelerating voltage of 120 kV and LaB_6_ as electron source.

X-ray diffraction (XRD) analysis was carried out at in continuous-scanning mode between 30° and 85° 2θ angle with a scanning rate of 0.00013° s^−1^ (step size of 0.002° 2θ and 150 s as counting time) (X’Pert PRO diffractometer, PANalytical, Almelo, The Netherlands). The diffractograms were obtained directly on the 3D printed specimens (before post-curing) and acquired under the same experimental conditions.

Tensile tests until failure were carried out according to the standard ISO 527-2 and carried out by using a 2 kN load cell, at a crosshead speed of 1 mm/min, in environmental condition of temperature and relative humidity (Universal Testing Machine, TesT GmbH, Erkrath, Germany).

Dynamic-mechanical thermal analysis (DMTA) was carried out by TA 800Q DMA instrument (TA instruments, New Castle, DE, USA) equipped with a single-cantilever clamp. Suitable specimens with rectangular shape (5 × 2 × 30 mm^3^) were obtained from the narrow parallel-sided portion of the 3D printed dumbbell-shaped specimens. Dynamic storage modulus *E*′, loss modulus *E*″ and loss factor tan δ were recorded from −50 to 80 °C at a heating rate of 3 °C/min, at a controlled sinusoidal strain (0.1% as maximum strain) and with a fixed frequency (1 Hz).

The creep behavior of unfilled and filled 3D printed specimens was investigated by applying a constant stress (0.05 MPa) for a creep time of 10 min at several isothermal steps (temperature range from 10 to 50 °C, temperature increment 10 °C). Raw creep curves were used to generate a master curve at 10 °C expressed as creep compliance J_c_ as a function of time, according to the time-temperature superimposition (TTS) principle according to the Williams-Landel-Ferry (WLF) model [36].

To predict the reduced storage modulus, the generalized Kernel equation for the reduced modulus of filled polymers was applied and a comparison with the experimental results was carried out, using the following:

(1) $$\frac{E^{\prime }}{E_{1}^{\prime }}=\frac{1+AB\varphi_{2}}{1+B\Psi \varphi_{2}}$$

where *E′* and $E_{1}^{\prime }$ are the storage moduli of the composite and of the unfilled matrix, respectively. The constant *A*, for spherical particles, is defined as:

(2) $$A=\;\frac{7-5\nu }{8-10\nu }$$

being ν the Poisson’s ratio. The constant *B* depends on the ratio between the filler and the matrix moduli, but it can be approximated to 1 for very high ratios.

Ψ is a reduced concentration term which depends on the maximum packing fraction of the particles ($\varphi_{m}$) according to the following definition:

(3) $$\Psi =1+\frac{1-\varphi_{m}}{\varphi_{m}^{2}}\varphi_{2}$$

in which $\varphi_{2}$ represents the filler volume fraction. To convert the percentage weight values into volume fractions, the density of the AgNPs has been taken equal to that of the bulk silver (10.49 g/cm^3^), while for the matrix the density was taken equal to the one of the liquid resin formulation (1.127 g/cm^3^).

In this work, the Poisson’s ratio of the matrix was taken equal to 0.5 and the maximum packing fraction of the particles $\varphi_{m}$ was taken equal to 0.601, which is typical of the non-agglomerated packing configuration.

## 3. Results

### 3.1. XRD and TEM Analysis

**Figure 2:**
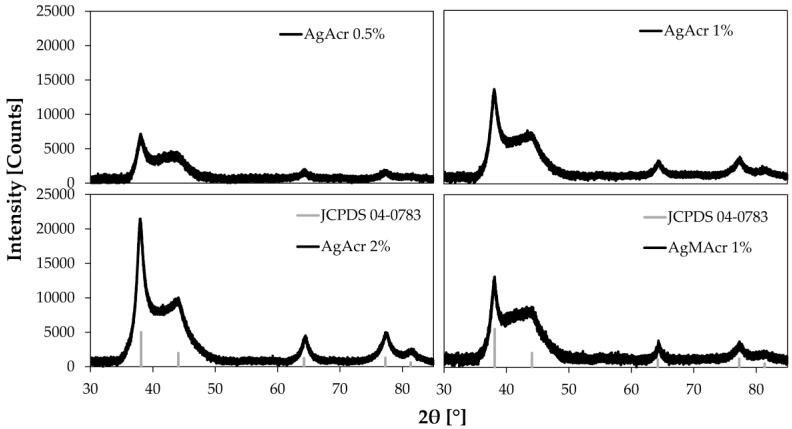
X-ray diffraction (XRD) diffraction pattern of 3D printed materials containing different amount of AgNPs from AgAcr and AgMAcr as precursors.

XRD patterns of “AgAcr *x*%” and “AgMAcr 1%” samples reported in Figure 2 confirm the transformation of silver ions to metallic silver. It is also important to underline the absence of peaks related to silver oxide (main peak at 32.9°). Therefore, the peak intensity decreases from “AgAcr 2%” to “AgAcr 0.5%” sample, according to the variation of the nanoparticle amount in the analyzed samples.

Moreover, the evident peak broadening is indicative of the nanometric dimension of the coherent diffraction domains.

**Figure 3:**
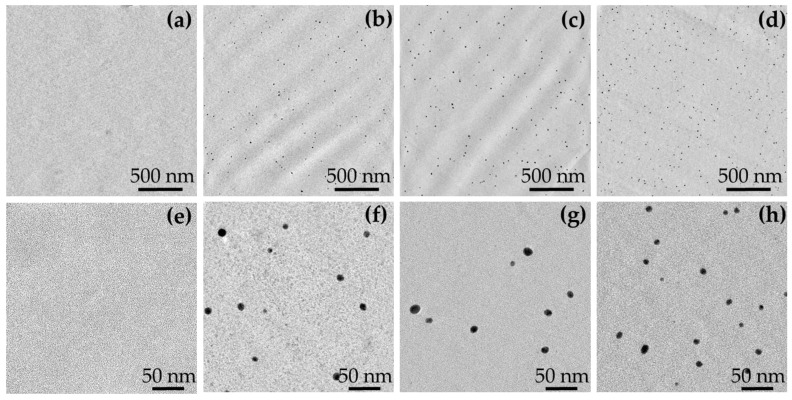
Transmission electron microscopy (TEM) micrographs at two different magnifications of “Unfilled” (**a**,**e**); “AgAcr 1%” (**b**,**f**); “AgAcr 2%” (**c**,**g**); and “AgMAcr 1%” (**d**,**h**); as representative.

Typical TEM micrographs of unfilled and filled resins are reported in Figure 3.

TEM micrographs show that, compared to the unfilled sample, AgNPs can be observed in all filled systems, regardless of the initial load and type of used AgNPs precursor. AgNPs present an almost spherical geometry with a narrow size distribution. The measured average dimensions are 9 ± 2, 13 ± 3, and 11 ± 2 nm in “AgAcr 1%”, “AgAcr 2%” and “AgMAcr 1%” samples, respectively.

### 3.2. DSC Analysis

**Figure 4:**
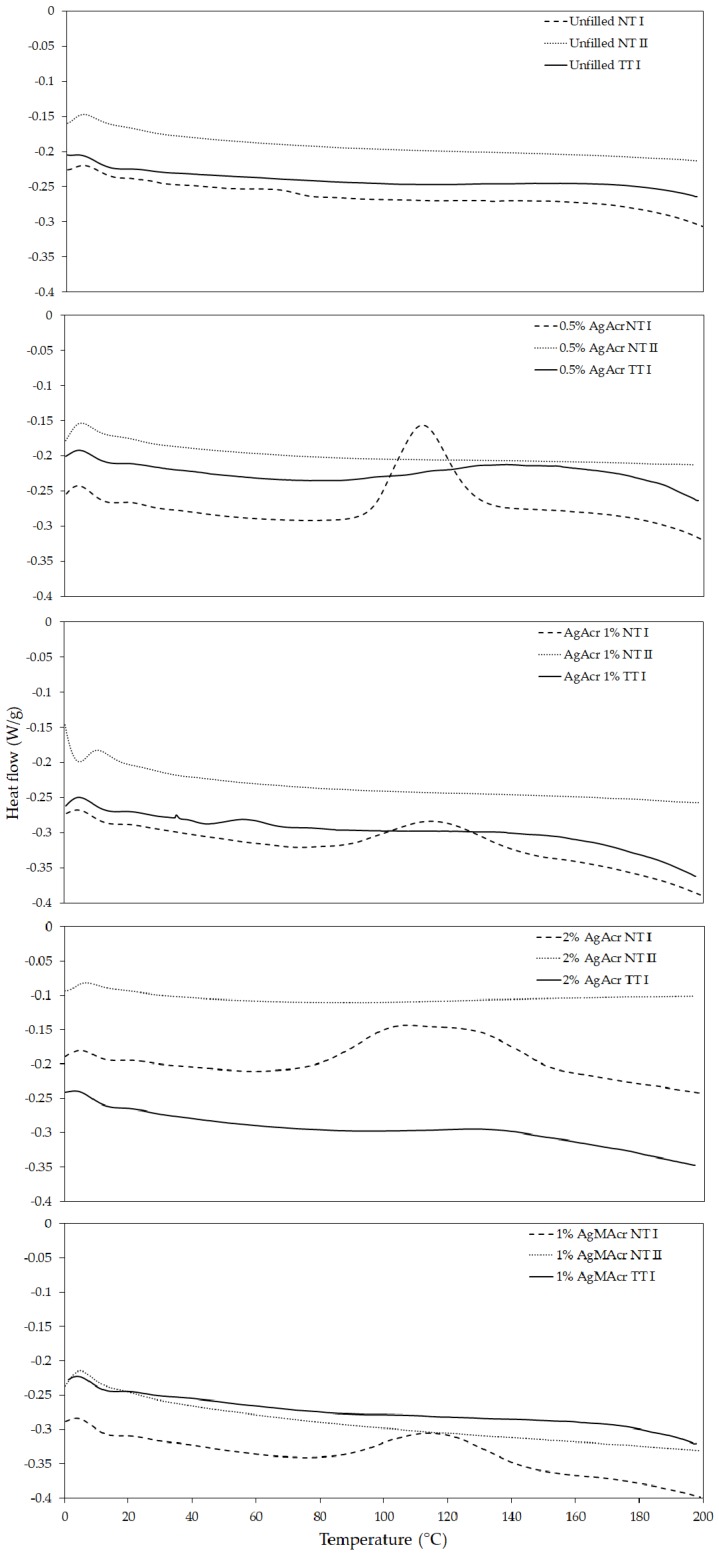
Differential scanning calorimetry (DSC) thermograms (hexo up) of “Unfilled”, “AgAcr *x*%” and “AgAcr 1%” (TT: after thermal treatment; NT: no thermal treatment; I: first heating scan, II: second heating scan).

Typical DSC thermograms of unfilled and filled resins, before and after a post-curing thermal treatment, are reported in Figure 4.

DSC thermogram of unfilled resins “Unfilled NT I” is characterized by the absence of any exothermic peak indicating a complete resin conversion on the 3D printed specimens without the necessity of any post-curing processes. On the contrary, all filled resins, characterized immediately after 3D printing (NT I samples), exhibit clear exothermic peaks attributable to the presence of unreacted carbon-carbon double bonds. These peaks completely disappear in the second heating scan (NT II samples) and are absent in the thermally post-cured samples (TT I samples).

### 3.3. Tensile Properties

**Figure 5:**
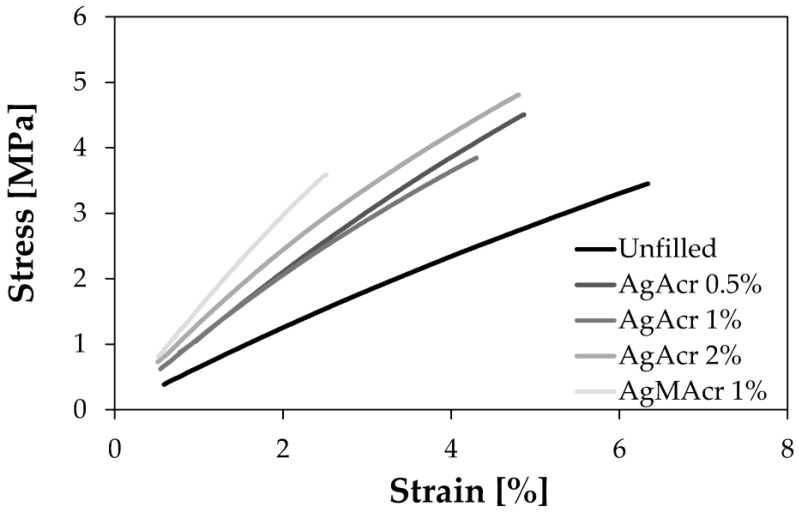
Representative stress-strain diagram for Ag-filled and unfilled samples.

Representative stress-strain curves of filled and unfilled resins are reported in Figure 5. The corresponding tensile properties of the 3D printed specimens are reported in Table 1.

Young’s modulus values systematically increase by increasing the AgNPs concentration from a minimum of 68 MPa for “Unfilled” sample to a maximum of 153 MPa for “AgMAcr 1%” sample. Also strength values follow the same trend with a significant maximum value of 5.0 MPa in the case of “AgAcr 2%” sample.

Strain at break values systematically decrease by increasing the AgNPs concentration from a maximum of 6.1% for “Unfilled” sample to a minimum of 2.4% for “AgMAcr 1%” sample.

### 3.4. Dynamic-Mechanical Thermal Properties

The results obtained by dynamic-mechanical analysis are reported in Table 2 in terms of glass transition temperature (*T*g_DMTA_, determined from the maximum of tanδ curve) and storage modulus measured at 60 °C.

*T*g_DMTA_ values systematically increase by increasing the AgNPs concentration from a minimum of 0.7 °C for “Unfilled” sample to a maximum of 11.8 °C for “AgAcr 2%” sample. Also storage modulus values evaluated above *T*g_DMTA_ follow a similar trend ranging from a minimum of 65 MPa for “Unfilled” sample to a maximum of 140 MPa for “AgMAcr 1%” sample.

**Figure 6:**
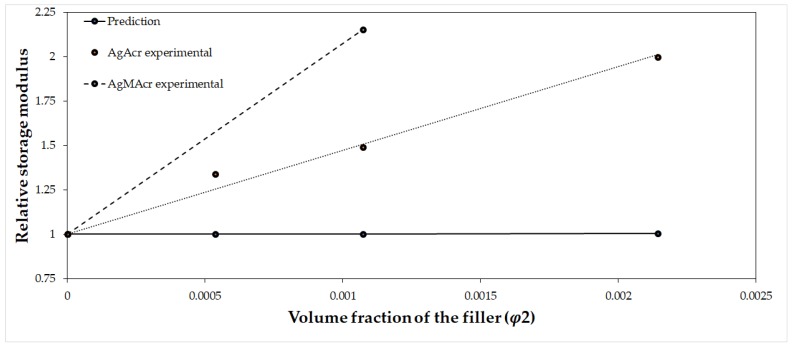
Relative storage modulus as a function of AgNPs volume fraction. A comparison between predicted and experimental values for the AgAcr filled series.

A comparison between the reduced storage modulus obtained from the experimental results and from predictive equations is reported in Figure 6 by applying the generalized Kernel equation for the reduced modulus of filled polymers [37].

The figure clearly shows an evident increment of the relative storage modulus (*E*′*/E*_1_′) in the experimental results with respect to the prediction of the Kernel equation. This increment can be attributed to an action of the filler as a cross-linking agent, besides his action of pure rigid particle reinforcement.

### 3.5. Creep Properties

**Figure 7:**
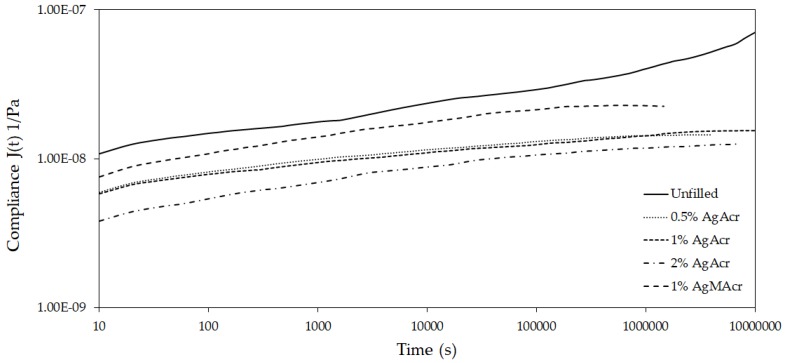
Master curves of compliance as a function of time at the reference temperature of 10 °C for “Unfilled”, “AgAcr *x*%”, and “AgMAcr 1%” samples.

Creep data (compliance as a function of temperature) obtained at several isothermal conditions ranging from 10 to 85 °C were elaborated by applying the time-temperature superimposition principle in order to get information on the creep behavior of the investigated materials in a time scale much higher than the experimentally accessible one. A portion of the obtained master curves at the reference temperature of 10 °C is reported in Figure 7.

All compliance values were in the range of 10^−8^ Pa^−1^ but there was a clear difference between filled and unfilled resins notwithstanding a similar slope of the curves. Once again the materials containing in situ generated AgNPs presented a more elastic behavior here represented in terms of creep resistance.

## 4. Discussion

TEM analysis showed that the in situ formation of AgNPs during 3D printing allowed to optimize the dispersion and distribution of the nanofillers within the polymer matrix, avoiding problems related to the aggregation phenomena usually observed in the alternative ex situ preparative approaches.

DSC analysis indicated an uncomplete polymerization reaction in the case of filled resins, i.e., in the presence of silver salts as AgNPs precursors. Presumably, two different phenomena can contribute to a conversion of carbon-carbon double bonds lower than 100%. First of all, the in situ generation of AgNPs converts the materials from a substantially homogeneous system to a bi-phasic heterogeneous one with the two phases having different refractive indexes, which in turn could lead to light scattering phenomena with a decrease of photo-polymerization reaction efficiency. Secondly, the presence of rigid AgNPs in the polymer matrix is also expected to increase the rigidity and the glass transition temperature of the material and an earlier vitrification could occur with a stop of the chemical reactions due to limitation in the diffusion phenomena within a glassy matrix.

XRD analysis confirmed the conversion of silver cations deriving from soluble silver salts to metallic silver thanks to the occurrence of a reduction process attributable to both UV radiation (from SLA printer) and presence of radicals, formed after dissociation of photo-initiator. This is a very important result taking into account that similar systems processed with an alternative technology such as Digital Light Processing (DLP) evidenced the necessity to a UV-based post-curing step to generate AgNPs [21,38]. From this point of view, the power (light intensity) of the UV radiation used in SLA process was high enough to activate the chemical reduction processes to in situ generate AgNPs during the 3D printing step, contrary to what observed by using a different additive manufacturing technology (i.e., DLP) characterized by a less intensive radiation.

The increment in Young’s modulus, due to the presence of a rigid filler, is an expected result due to hydrodynamic effect deriving from the inclusion of rigid particles in a less rigid polymer matrix. On the other hand, data indicates that the cross-linking density, which in turn dominates the modulus value above *T*g, was not negatively affected by the in situ generation of AgNPs and related by-products formation. Moreover, the use of silver salts with counter-anions which can react directly with the acrylic monomers during the formation of the polymer network, can further stabilize the final system, limiting the formation of extractable by-products.

In addition to the significant increment of Young’s modulus evidenced for all the filled samples (with a remarkable maximum value of 153 MPa for “AgMAcr 1%”), it is noteworthy also the increment of strength in the case of “AgAcr *x*%” series with respect the unfilled reference resin (with a maximum value of 5.0 MPa with respect to 3.4 MPa). This behavior is quite unexpected taking into account that strength usually decreases by increasing the filler content in the case of micro-composites. Conversely, in the present case a positive contribution can be considered as deriving by both the nanometric size of the filler combined with an enhanced particle/matrix interfacial adhesion according to other similar evidences reported in literature [39]. In this case, the interactions at the nanoparticle-polymer interface can benefit from the presence of carboxylate groups present in the polymeric network and deriving from the counter-anion of the starting salt. In fact, the acrylate and methacrylate groups are characterized by the co-existence of alkenyl and carboxylic functionalities in the same molecule. The alkenyl functionality has the possibility to react with the acrylic monomers, becoming part of the polymeric structure. This behavior determines the formation of carboxylic pendants, scattered along the polymer chain, which can coordinate the surface of the nanoparticles, improving the interactions at the interface between AgNPs and polymer.

As expected, the presence of a high-modulus filler resulted in a decrement of strain at break for all the filled resins, even if the loss in strain at break was relatively limited, with respect to the reference value of 6.1% for unfilled resin.

Accordingly to quasi-static tensile properties above reported, also DMTA and creep data indicated a significant increment of rigidity, proportional to the amount of AgNPs as indicated by both glass transition temperature, storage modulus and compliance values. The comparison between experimental and predicted storage modulus values further supported the presence of an enhanced interfacial AgNPs-polymer adhesion, as already evidenced in previous papers [40,41,42].

## 5. Conclusions

This article demonstrates that the SLA process can simultaneously reduce a silver salt to an in situ generate well dispersed and distributed AgNPs while polymerizing the liquid monomer into a solid matrix. Thanks to the selection of silver salts having reactive counter-ions, such as acrylate and methacrylate, the obtained nanocomposite material is homogeneous and without extractable by-products. In fact, the reactive counter-ions can become part of the polymeric structure and stabilize the nanoparticle-polymer interfacial interactions. The absence of by-products release, the presence of AgNPs with good dispersion and distribution, the freedom and precision allowed in the fabrication by the SLA process, are all factors that can promote this approach for the realization of useful and valid nanocomposite materials applicable to various sectors ranging from medical and healthcare, to food packaging.

## Figures and Tables

**Table 1:** Tensile properties of 3D printed specimens (*E*_t_: Young’s modulus; σ_M_: tensile strength; ε_B_: tensile strain at break, according to ISO 527 technical standard).

Sample	*E*_t_ (MPa)	σ_M_ (MPa)	ε_B_ (%)
Unfilled	68 ± 2	3.4 ± 0.3	6.1 ± 0.7
AgAcr 0.5%	111 ± 3	4.3 ± 0.4	4.8 ± 0.6
AgAcr 1%	113 ± 8	4.0 ± 0.4	4.0 ± 0.1
AgAcr 2%	142 ± 8	5.0 ± 0.8	4.7 ± 0.9
AgMAcr 1%	153 ± 5	3.4 ± 0.3	2.4 ± 0.2

**Table 2:** Glass transition temperature (*T*g_DMTA_, evaluated as temperature value corresponding to tanδ peak) and storage modulus at 60 °C values for all the printed specimens, from Dynamic-mechanical thermal analysis (DMTA) analyses.

Sample	*T*g_DMTA_ (°C)	*E*′ at 60 °C (MPa)
Unfilled	0.7	65
AgAcr 0.5%	4.9	87
AgAcr 1%	5.1	97
AgAcr 2%	11.8	130
AgMAcr 1%	10.5	140
